# The Practice of Home Visiting by Community Health Nurses as a Primary Healthcare Intervention in a Low-Income Rural Setting: A Descriptive Cross-Sectional Study in the Adaklu District of the Volta Region, Ghana

**DOI:** 10.1155/2021/8888845

**Published:** 2021-03-24

**Authors:** Kennedy Diema Konlan, Nathaniel Kossi Vivor, Isaac Gegefe, Imoro A. Abdul-Rasheed, Bertha Esinam Kornyo, Isaac Peter Kwao

**Affiliations:** ^1^Department of Public Health Nursing, School of Nursing and Midwifery, University of Health and Allied Sciences, Ho, Ghana; ^2^College of Nursing, Yonsei University, 50-1, Yonsei-ro, Seodaemun-gu, Seoul 03722, Republic of Korea

## Abstract

**Background:**

Home visit is an integral component of Ghana's PHC delivery system. It is preventive and promotes health practice where health professionals render care to clients in their own environment and provide appropriate healthcare needs and social support services. This study describes the home visit practices in a rural district in the Volta Region of Ghana. *Methodology*. This descriptive cross-sectional study used 375 households and 11 community health nurses in the Adaklu district. Multistage sampling techniques were used to select 10 communities and study respondents using probability sampling methods. A pretested self-designed questionnaire and an interview guide for household members and community health nurses, respectively, were used for data collection. Quantitative data collected were coded, cleaned, and analysed using Statistical Package for Social Sciences into descriptive statistics, while qualitative data were analysed using the NVivo software. Thematic analysis was engaged that embraces three interrelated stages, namely, data reduction, data display, and data conclusion.

**Results:**

Home visit is a routine responsibility of all CHNs. The factors that influence home visiting were community members' education and attitude, supervision challenges, lack of incentives and lack of basic logistics, uncooperative attitude, community inaccessibility, financial constraint, and limited number of staff. Household members (62.3%) indicated that health workers did not adequately attend to minor ailments as 78% benefited from the service and wished more activities could be added to the home visiting package (24.5%).

**Conclusion:**

There should be tailored training of CHNs on home visits skills so that they could expand the scope of services that can be provided. Also, community-based health workers such as community health volunteers, traditional birth attendants, and community clinic attendants can also be trained to identify and address health problems in the homes.

## 1. Introduction

Home visit practice is a healthcare service rendered by trained health professionals who visit clients in their own home to assess the home, environment, and family condition in order to provide appropriate healthcare needs and social support services. The home environment is where health is made and can be maintained to enhance or endanger the health of the family because individuals and groups are at risk of exposure to health hazards [1, 2]. At home visit, conducted in a familiar environment, the client feels free and relaxed and is able to take part in the activity that the health professional performs [1]. It is possible to assess the client's situation and give household-specific health education on sanitation, personal hygiene, aged, and child care. The important role the health professional plays during home visits (HV) cannot be overemphasized, and this led Ghana to adopt HV as a cardinal component of its preventive healthcare delivery system. This role is largely conducted by community health nurses (CHN) [2]. Health education given during HVs is more effective, resulting in behavioural change than those given through other sources such as the mass media [3].

In the home, the health professionals, mostly CHN monitor the growth, development, and immunization status of children less than 5 years and carry out immunization for defaulters. Care is given to special groups such as the elderly, discharged tuberculosis, and leprosy patients as well as malnourished children [1, 2]. It is also possible to carry out contact tracing during HVs [2]. These services may prevent, delay, or be a substitute for temporary or long-term institutional care [4, 5]. HV has potential for bringing health workers into contact with individuals and groups in the community who are at risk for diseases and who make ineffective or little use of preventive health services [2]. Several factors influence the conduct of HVs. These factors include location of practice, general practitioners age, training status, and the number of older patients on the list and predicts home visiting rate [6].

The concept of HV has remained in Ghana over the decades, and yet, its very essence is imperative [3]. In Ghana, home visiting is one of the major activities of CHN. The health visitors, as CHNs were then called, went from house to house, giving education on sanitation and personal hygiene [3]. These nurses attempt to promote positive health and prevent occurrence of diseases by increasing people's understanding of healthy ways of living and their knowledge of health hazards [7]. HVs remain fundamental to the successful prevention of deaths associated with women and children under five; yet, there still remain certain gaps in the successful implementation of this innovative intervention in Ghana [4]. In Sekyere West district in Ashanti Region of Ghana, although nurses had knowledge of home visiting and had a positive opinion of the practice, they could not perform their home visiting tasks or functions up to standard [8]. Home visiting practice in that district among nurses was found to be very low, even though community members desired more [8]. The findings indicate that there is a need for HV [9]. Also identified were several health hazards, such as uncovered refuse containers, open fires, misplaced sharp objects, open defecation, and other unhygienic practices that a proper home visiting regiment can address [8]. At the service level, lack of publicity about the service, the cost of the service, failure to provide services that meet clients' felt needs, rigid eligibility criteria, inaccessible locations, lack of public transport, limited hours of operation, inflexible appointment systems, lack of affordable child care, poor coordination between services, and not having an outreach capacity were identified as the challenges associated with this kind of service [9–13].

Home visiting is a crucial tool for enhancing family healthcare and the health of every community. Ghana Health Service through home visiting services has supported essential community health actions and address gaps in knowledge and community practices such as reproductive behaviour, nutritional support for pregnant women and young children, recognition of illness, home management of sick children, disease prevention, and care seeking behaviours [4]. As many interventions are implemented by stakeholders in health to ensure that home visiting practices actually benefit community members, recent studies have not delved into the practices of home visiting in poor rural communities especially in the Volta Region of Ghana. This study assessed the home visiting practices in the Adaklu district (AD) of the Volta Region.

### 1.1. Aim

This study assessed the practice of home visiting as a primary healthcare (PHC) intervention in a poor rural district in the Volta Region of Ghana.

## 2. Methodology

### 2.1. Study Design

This mixed method study employed a descriptive cross-sectional study design as the study involved a one-time interaction with the CHNs and the community members to assess the practice of HVs.

### 2.2. Study Setting

The AD is one of the districts in the Volta Region of Ghana and has about 40 communities. The district capital and administrative centre is Adaklu Waya. The estimated population of the district was 36391 representing 1.7% of the Volta Region's population before the Oti Region was carved out [14]. The district is described as a rural district [14] as no locality has a population above 5000 people. The economically active population (aged 15 and above) represents 67% of the population [14]. The economically inactive population is in full-time education (55.1%), performed household duties (20.6%), or disabled or too sick to work (4.6%), while the employed population engages in skilled agricultural, forestry, and fishery workers (63.1%), service and sales (12.6%), craft and related trade (14.6%), and 3.4% other professional duties [14]. The private, informal sector is the largest employer in the district, employing 93.9% [14]. There are 15 health facilities in the district government health centres [4], one health centre by Christian Health Association of Ghana, and 10 community health-based planning services (CHPS) of which 5 are functional [15]. The housing stock is 5629 representing 1.4% of the total number of houses in the Volta Region. The average number of persons per house was 6.5 [14], and the houses are mostly built with mud bricks [15]. The most common method of solid waste disposal by households is public dumping in the open space (47.5%). Some households dump solid waste indiscriminately (17.3%), while other households dispose of burning (13.3%) [14].

### 2.3. Study Population, Sample, and Sampling Technique

There are about 36391 inhabitants with 6089 households in AD [14]. This study mainly involved adult members of the household and CHNs from randomly sampled communities in the district. These sampled communities included Abuadi, Anfoe, Ahunda, Dawanu, Goefe, Helekpe, Hlihave, Tsrefe, Waya, and Wumenu. An adult member of the household is a person above the age of 18 years who has the capacity to represent the household. CHN [11] from the selected communities in the district was recruited. A CHN is a certified health practitioner who combines prevention and promotion health practices, works within the community to improve the overall health of the area, and has a role to play in home visiting.

Estimating for a tolerable error of 5%, with a confidence interval of 95%, and a study population of 6089 households, with a margin of error of 0.05 using Yamane's formula for calculating sample for finite populations, a sample of 375 households were computed. The sample size was increased to 390 to take into consideration the possible effect of nonresponse from participants. Multistage sampling technique was adopted to eventually select study participants. Each community was stratified into four geographical locations: north, south, east, and west with respondents being selected from every second house using a systematic sampling approach. In each household, an adult member of the household responded to the questionnaire.

A whole population sampling method was used to select eleven [11] CHNs from the specific communities [10] where the study took place in the district. The CHN that served the 10 selected communities were selected. The numbers selected from each community were Helekpe (18.2%), Waya (18.2%), Anfoe (9.1%), Tsrefe (27.3%) and Wumenu (27.3%). This represented 42.3% of the total CHN community of the district at the time of the study.

### 2.4. Pretesting

The questionnaire and interview guide were piloted using 30 adult household members and 5 CHNs, respectively, at Klefe CHPS in the Ho municipality. The data collected through the questionnaire were subjected to a reliability test on SPSS (version 22). The pretesting ascertained the respondent's general reaction and particularly, interest in answering the questionnaire. The questionnaire was modified until it produced a Cronbach alpha coefficient of 0.790. It can therefore be concluded that the questionnaire had a high reliability in measuring the objectives of the study. The pretesting helped in identifying ambiguous questions and revising them appropriately. It also helped to structure and estimate the time the respondents used to answer the questionnaires and to respond to the interview.

### 2.5. Data Collection

Researchers from the University of Health and Allied Sciences School of Nursing and Midwifery were involved in data collection. Five researchers received two days training in data collection, the study tools, and research ethics for social sciences prior to the commencement of data collection. All researchers had a minimum of a bachelor degree in CHN with at least three years' data collection experience.

Respondents were assisted to respond to a questionnaire within their homes. The household questionnaire had four [4] sections comprising personal details and how HV practice is carried out in the home such as frequency of visit, duration, and activities. Subsequent sections had respondents answer questions on the challenges, benefits, and factors that could promote the HV practice. It took an average of about 15 minutes to complete a single questionnaire.

A semistructured interview guide was used to interview CHNs. This guide was in four sections; the first section was personal details with subsequent sections on practice of home visits, constraints to the practice, the benefits, and promotion factors to HVs. An interview section lasted 20–25 minutes to complete.

### 2.6. Data Analysis

#### 2.6.1. Quantitative Data

Each individual questionnaire was checked for completeness and appropriateness of responses before it was entered into Microsoft Excel, cleaned, and transferred to the Statistical Package for Social Sciences (version 22) for analysis. The data were basically analysed into descriptive statistics of proportions. There were also measures of central tendencies for continuous variables.

#### 2.6.2. Qualitative Data

In data analysis, thematic analysis was engaged that embraces three interrelated stages, namely, data reduction, data display, and data conclusion [16]. CHNs views were summarised based on the conclusions driven and collated as frequencies and proportions. Guest, Macqueen, and Namey summarised the process of thematic analysis as construing through textual data, identifying data themes, coding the themes, and then interpreting the structure and content of the themes [17]. In using this scheme, a codebook was first established, discussed, and accepted by the authors. The nodes were then created within NVivo software using the codebook. Line-by-line coding of the various transcripts was performed as either free or tree nodes. Double coding of each transcript was carried out by two of the researchers. Coding comparison query was used to compare the coding, and a kappa coefficient (the measurement of intercoder reliability) was generated to compare the coding that was conducted by the two authors. The matrix coding query was performed to compare the coding against the nodes and attributes using NVivo software that made it possible for the researchers to compare and contrast within-group and between-group responses.

### 2.7. Ethical Consideration

Ethical clearance was obtained on the 19th September, 2018, from the Research and Scientific Ethics Committee of the Institute of Health Research, University of Health and Allied Sciences (UHAS-REC A.2 [13] 18-19). Permission was sought from the district health authorities, chiefs, and assembly members of each study community. Preliminary to the administration of the questionnaires, an informed consent was obtained as respondents signed/thumb printed a consent form before they were enrolled into the study. Participants could withdraw from the study anytime they wished to do so.

## 3. Results

### 3.1. Household Members' Views regarding Home Visit

The household representatives surveyed (375) had a mean age of 41.24 ± 16.88 years. The majority (26.5%) of household members were aged between 30 and 39 years. Most (75.1%) were females. The majority (97.1%) of people in households were Christians, while 38% was farmers. The majority (69.9%) of household members were married as 47.2% had schooled only up to the JHS level as at the time of this survey as given in Table 1.

The majority (73.3%) of adult household members had ever been visited by a health worker for the purpose of conducting HVs as a significant number (26.7%) of household members had never been visited by health workers in the community. Most (52.6%) household members had had their last visit from a health worker during the past month. Within the past three months, some (48.2%) community members were visited only once by a health worker. The majority (93.4%) of community members were usually visited between the time periods of 9am and 2pm as given in Table 2. The community members contend that home visiting was beneficial to the disease prevention process (65%). The people that need to be visited by CHNs include children under five (25%), malnourished children's homes (14%), children with disabilities (14%), mentally ill people (11%), healthcare service defaulters (22%), people with chronic diseases (9%), and every member of the community (5%).

Most (87.9%) community members were given health education during HVs conducted by the CHN. In describing the nature of health education that is most frequently given by CHNs during HVs, household members indicated fever management (14%), malaria prevention (20%), waste disposal (11%), prevention and management of diarrhoea (22%), nutrition and exclusive breastfeeding (14%), hospital attendance (14%), and prevention of worm infestations (5%). The majority (62.3%) of community members did not receive a minor ailment management during HVs as most (66.5%) of community members received vaccination during HVs by CHNs. Describing the type of minor ailment treatment given during the HV include care of home accidents (13%), management of minor pains (22%), management of fever (45%), and management of diarrhoea (20%). Household members (24.5%) did identify bad timing as a barrier for home visiting, while some (13.1%) did identify the attitude of health workers as a barrier to home visiting. However, most (67.3%) of the household members attributed their dislike for home visiting to the duration of the visit. The majority (95.2%) of household members indicated health workers were friendly. Some household members (78%) indicated they benefited from HVs conducted in their homes. The majority (91.4%) of household members showed that time for home visiting was convenient. Indicating if household members will wish for the conduct of the HV to be a continuous activity of CHNs in their community, the respondents (82%) were affirmative.

### 3.2. CHNs Views on Home Visit in AD

The mean age of CHNs was 30.44 ± 4.03 years as some (33.3%) were aged 32 years as the modal age. The CHNs (90.9%) were females with the majority (81.8%) being Christians as given in Table 3.

In assessing the home visiting practices of CHNs, the researchers had some thematic areas. These thematic areas that were discussed include but not limited to the concept of HV by CHN, factors that influence the conduct of HVs, ability to visit all homes within CHN catchment area, reasons for conducting or not able to conduct HV, frequency of conducting home visits by CHN, and activities undertaken during HVs. This view that was expressed was simply summarised based on the thematic areas and presented in Table 4 as descriptive statistics related to the CHN conduct of HVs.

#### 3.2.1. Concept of Home Visit by CHN

CHNs have varied descriptions of the concept of HV as it is conducted within the district. The description of HV was basically related to the nature and objective that is associated with the concept. The central concept expressed by participants included a health worker visiting a home in their place of abode or workplace, providing service to the family during this visit, and this service is aimed at preventing disease, promoting health, and maintaining a positive health outcome. These views were summarised when they said

“HVs are a service that we (CHNs) rendered to the client and his family in their own home environment to promote their health and prevent diseases. The central idea is that during the HV, the CHN is able to engage the family in education and services that eventually ensure that diseases are prevented and health is promoted.”

“HV is the art when the CHNs visit community members' homes to provide some basic curative and largely preventive healthcare services to clients within their own homes or workplaces. During this visit, the CHN helps the entire family to live a healthy life and give special attention or care to the vulnerable members of the society.”

“It is the processes when at-risk populations are identified; then, the CHN provides services to this cadre within their own home environment and sometimes workplaces as the case may be. Essentially, the CHN assists the family to adopt positive behaviours that will ensure they live with the vulnerable person in a more comfortable way.”

#### 3.2.2. Factors that Influence the Conduct of Home Visits

The CHNs enumerated a cluster of factors that influence the conduct of HVs within the district. These factors ranged from community members education, attitude, supervision challenges, lack of incentives, and lack of basic logistics to conduct HVs. The uncooperative attitude of community members was identified by CHNs (36.4%) as a barrier to HVs. As they indicate, some community members did not support the continued visit to their homes or did not give them the necessary attention needed for the provision of services.

“Some community members do not understand the importance of HVs in the prevention of disease and for that matter are less receptive to the conduct of HVs. They just do not see the need for the service provider to come to their homes to provide services.”

“The client is the master of his own home; when you get into a home for a HV, the owner should be willing to talk or attend to you. Sometimes, you get into a home and even if you are not offered a seat, or you are just told we are busy, come next time. You know community service is not a paid job, so because the community members do not directly pay for the services we provide, essentially less premium is placed on the activities we conduct.”

“There is some resistance to HVs by some community members. Sometimes, you come to a house and can feel that you are not wanted; meanwhile, the home is part of the home that needs and has to get a HV because of the special needs they have. This is particularly specific in homes that believe that the particular problem is a result of supernatural causes.”

#### 3.2.3. The Ability to Visit All Homes within CHN Catchment Area

The conduct of HVs is a basic responsibility for all CHNs as they remain as an integral part of the PHC delivery system in Ghana. Based on the nature and problems in the community, CHNs strategizes various means that will aid them to provide this essential service efficiently. CHNs (81.8%) are able to visit all homes in the catchment areas during a quarter. Some of the responses included the following:

“We do organise HVs, this is part of our routine schedule. As a community health nurse, to enjoy your work, you will need to organise HVs from time to time.”

“As for the HV, it depends on the strategies a particular CHPS compound is using. Irrespective of the community that one works in, you can always provide full and adequate care and service to the community if you plan well. First, you have to identify the “at need people” then the distance to their homes and put this in your short-term strategic plan for execution.”

“HVs are basic responsibilities of community health nurses, and we ought to execute it. In spite of the challenges, we cannot let those particularly hinder on our ability to conduct our very core mandate.”

Some CHNs were not able to visit all homes in their catchment areas, citing “hard to reach areas” and “Inadequate equipment” as the reasons for not being able to visit all households.

“Sometimes it is the distance to the clients' homes that makes it impossible to visit them. There are some homes if you actually intend to visit them, then you must be willing to spend the whole day doing only that activity.”

“Some clients' problems are such that you will need to have special tools before you visit them. For example, what use will it be to a diabetic client if you visit him/her and you are unable to monitor the blood sugar level or to a hypertension patient, you are not able to check the blood pressure because you do not have the required equipment?”

“To have a successful HV practice, I think the authorities should be willing to provide the basic logistics that will aid us to work. Without this basic logistics, we cannot.”

#### 3.2.4. The Reasons for Conducting or Not Able to Conduct Home Visits

CHNs (72.7%) carried out both routine and special HVs. For those community health nurses who were not able to conduct HVs, several reasons were ascribed. Some of the reasons described included the lack of basic amenities to conduct HVs. The majority (18.2%) of CHNs also did attribute inaccessible geographical areas as a barrier to HV. Also, CHNs (63.6%) identified inadequate logistics and financial constraints as barriers to HV. All of the CHNs report on their activities regarding home visiting to the district health authorities.

“We basically lack the simple logistics that will assist us to conduct HVs. We do not have simple movable equipment like weight scales, thermometers, sphygmomanometers, and stethoscopes.”

“We do not have functionally equipped home visiting bags, so even if we decide to visit the homes, how much help will we be to the client?”

The other reasons included large catchment areas and lack of reliable transportation for the conduct of HVs in the AD.

“The catchment area is quite wide and practically impossible to visit every home. Looking from here to the end of our catchment area is more than 5 kilometers, without a means of transport, one cannot be able to visit all those homes.”

“I remember in those days; community health nurses were given serviceable motor cycles to aid in their movement and especially the conduct of HVs. Today, since our motorbike broke down 5 years ago, it has since not been serviced, yet we are expected to conduct HVs.”

“To conduct home visits, whose money will be used for transportation? The meagre salary I earn? Or the families or beneficiaries of the service have to pay?”

“The number of staff here is woefully inadequate, we are only two people here, how can we do home visiting and who will be left in the facility to conduct the other activities. For this reason, we are not able to conduct HVs.”

CHNs tried to visit the homes at various times depending on the occupation of the significant other of the homes, so that they can provide services in the presence of the significant others. CHNs (63.6%) visit 6–10 homes in a week as 90.9% CHNs conduct HVs in the morning. The reasons given for conducting some HVs in the evenings included the following:

“This place is largely a farming community, most people visit their farms during the mornings, so if you visit the home in the morning, you may not meet the significant others of the vulnerable person to conduct health education.”

“We do HVs because of the clients, so anytime it is possible, we will meet them at home, we conduct the visits at that time. For me, even if the case is that I can only meet the important people regarding the client at night, I visited them at that time. For community health nursing work, it is a 24-hour work and we must be found doing it at all time.”

#### 3.2.5. Frequency of Conducting Home Visits by CHN

Various schedule periods were used based on health facilities for the purpose of HVs. Most (45.5%) conducted HVs three times in a week. CHNs (90.9%) had conducted HVs the week preceding the interview. Indicating that the last time HV was conducted, CHNs conducted a HV at least within the last week:

“HV is a weekly schedule in this facility; for every week, we have a specific person who is assigned to do HV just as all other activities that are conducted in this facility”.

“Yes, last week, we had a number of HVs; we made one routine HV and the other was a scheduled HV from a destitute elderly woman who was accused as a witch by some of her family members.”

Indicating if they sometimes get fatigued for conducting HVs weekly because of the limited number of staff, a community health nurse indicated that,

“I think it is about the plan we have put in place. There are about four people in this facility. We plan our activities that we all conduct HVs. In a month, one may only have one or two HVs, so it is unlikely that you will be fatigued in conducting HVs.”

“Yes, sometimes, it is really tedious, but we cannot let that be a setback. We have a responsibility to execute and we must be doing so to the best of our ability.”

#### 3.2.6. Activities Undertaken during Home Visits

CHNs conducted health education (90.9%), management of minor ailments (54.6%), and vaccination/contact tracing (63.6%) during HVs. Describing if they are able to conduct the management of small ailments and home accidents at home, CHNs were divided in their ability to do this. Those were not able to do so indicated,

“…. And who will pay? Since the introduction of the national health insurance, we are not able to provide management of minor ailments during HVs. In those days, we were supplied with the medicines to use from the district, so we could provide such free services. But with the insurance now in place, we do not get medicine from the district, so whose medicine will you use to conduct such treatment?”

“I think our major goal is on preventive care. We have a lot to do with preventing diseases. Let us leave disease treatment to the clinical people. When we get ailments, we refer them to the next level of care to use their health insurance to access service.”

Identification of cases, defaulter tracing, and health education were identified as benefits and promotion factors of HVs. Identification of cases and defaulter tracing were both mentioned by CHNs as benefits and promotion factors of HVs.

“I think HVs should be continued and encouraged to be able to achieve universal, sustainable PHC coverage for all. Not only do we visit the homes, we also identify vaccination defaulters, tuberculosis treatment defaulters, and prevention of domestic violence against women and children and health education on specific diseases and sometimes we do immunisation.”

“In the home, we have a varied responsibility, treatment of minor ailments, immunization and vaccination, contact tracing, education on prevention of home accidents, etc.” It will be a disservice, therefore, if anyone tries to downplay the importance of HVs in our PHC dispensation.”

“Through HVs, we have provided very essential services that cannot be quantified mathematically, but the community members know the role of the services in their everyday lives. Even the presence of the community health nurse in the home is a factor that promotes girl child education and leads to woman empowerment.”

## 4. Discussion

This study assessed the home visiting practices in the AD of the Volta Region of Ghana. The concept of home visiting has been enshrined in Ghana's health history and executed by the CHN or public health nurses (PHN). In AD, only CHNs among all the various cadres of health professionals conducted HVs. This was contrary to the practice in the past when both CHN and PHN conducted HVs [18]. Notwithstanding the limited numbers of CHNs in the district, the majority of households (73.3 %) have a history of visits from a CHN. Home visiting is central in preventive healthcare services, especially among the vulnerable population. In children under five years, it is plausible that nurse home visiting could lead to fewer acute care visits and hospitalization by providing early recognition of and effective intervention for problems such as jaundice, feeding difficulties, and skin and cord care in the home setting [19]. Home visiting emphasizes prevention, education, and collaboration as core pillars for promoting child, parent, and family well-being [20].

In Ghana, under the PHC initiative, communities are zoned or subdivided and have a CHN to manage each zone by conducting HVs, including a cluster of responsibilities mainly in the preventive care sectors [4]. As rightly identified, HV is one of the core mandates of the CHN. Most of the community members who had received more than one visit in a week lived close to the health facilities indicating that there are homes which have never been visited, and CHNs are not able to cover all homes in their catchment areas. Factors that deter the conduct of HVs by CHN ranged from community members' level of education, attitude, supervision challenges, lack of incentives, and lack of basic logistics to conduct HVs. It is imperative that CHNs HVs especially those with newborn children to assess the home environment and provide appropriate care interventions and education as it was reported that 2.8% of 2641 newborns who did not receive a HV were readmitted to the hospital in the first 10 days of life with jaundice and/or dehydration compared with 0.6% of 326 who did receive a HV [21]. CHNs need to be provided with the right tools including means of transport to reach “hard to reach” communities and homes to provide services.

In rural Ghana such as the AD, community members leave the home to their places of work or farms during the morning sessions and only return home in the evening or late afternoon. HVs (93.4%) were conducted between 9am and 2pm, while some homes (6.6%) were visited between 3pm and 6pm. One problem faced by this timing difference is further expressed when CHNs indicated that they did not meet people at home during HVs. It is important for CHNs to be wary of their safety in client's homes as they show enthusiasm to visit homes at any time, and they could meet significant others. Therefore, to ensure safety, it is important to cooperate with clients and their families [22] in providing these services especially outside the conventional working hours. The need to use alternative timing of visits is essential as it is known that client participation is required to determine the scope of quality and safety improvement work; in reality, it is difficult for them to participate [23]. Also, some respondents indicated the time spent during HVs was too short (32.7%), and others (24.5%) wished the CHNs could spend more time with them. Community members have problems they wished could be addressed by the CHNs during HVs, but because of the number of households compared to the limited number of CHNs available, the CHNs could not spend much time during HVs and the respondents were not satisfied with the services rendered. It is likely that services will be better implemented by households if the CHN spends much time with the household and together implements thought health activities. Amonoo-Lartson and De Vries reported that community clinic attendants who spent more time in consultation performed better [24].

CHNs (8.2%) indicated they could not visit all households that needed the home visiting services in their catchment areas. Home visiting nurses are required to be mindful of the time and environment where they are performing care [22], so that they can allow for maximum benefit to the community. This notwithstanding, some community members (26.7 %) were not available during the HVs. The determination of suitable time between the CHN and the client is critical in ensuring that a positive relationship is established for their mutual benefit. The interval associated with HVs varied from one community or a health centre to another, and this was planned based on the specific needs of each community or CHPS catchment zone. There is actually no one-size-fits-all approach to home visiting [20] as several strategies can be adopted in providing services. The number of weeks or months elapsing between the visits ranged from one week to four months. The ministry of Health Ghana per the PHC system encourages CHN to conduct at least one contact tracing and/or HV session within a week within their communities [25]. All CHNs indicated that in their catchment area, they conducted at least one HV in a week and sometimes even more depending on the exigencies of the time.

Various activities are expected to be conducted by CHNs during HVs. These activities include the provision of basic healthcare services such as prevention of diseases and accidents, disease surveillance, tracing of contacts of infectious disease, tracing of treatment defaulters such as tuberculosis, diabetes mellitus, and hypertension and management of minor ailments at home. Community members (62.3%) did not receive a minor ailment management during HVs. CHNs are expected to be equipped with requisite knowledge, tools, and skills to be able to conduct these services in the homes. Also, the level of care that can be identified as a minor ailment as per the guidelines of the Ministry of Health needs to be specific as community members had varied classification of minor ailments and the level of care to be provided. Home visitors have varying levels of formal education and come from a variety of educational backgrounds marked by different theoretical traditions and content knowledge [20]. Other jurisdiction HV nurses drew blood for bilirubin checks and set up home phototherapy if indicated; they provided breastfeeding promotion and teaching on feeding techniques and skin and cord care [19]. Also, CHNs are expected to be able to provide baby friendly home-based nursing care services during a visit to the clients' home. HV nurses should also discuss the schedule of well-baby visits and immunizations [19] with families.

Important challenges associated with the conduct of HVs were identified as a large catchment area, lack of basic logistics, lack of the reliable transportation system, uncooperative community members, inadequate staff, and “hard to reach” homes due to geographical inaccessibility. Health education, management of minor ailment, and vaccination or contact tracing were the activities carried out in the homes. Home visiting nurses are under pressure to complete a job within an allotted time frame, as determined by the contract or terms of employment [22]. Time pressure significantly contributes to fatigue and depersonalization, and adjustments to interpersonal relationships with nurse administrators can have notable alleviating effects in relation to burnout caused by time pressure [26]. CHNs (63.6%) identified inadequate equipment and financial constraints as challenges to HV. Given evidence suggesting that relationship-based practices are the core of successful home visiting [27–29], with a natural harmony between the home visitor and the community members to the home, she renders her services [20]. A report published by the National Academy of Sciences (1999) also identified staffing, family involvement, language barrier, and cultural diversities as some of the barriers to a HV [30].

Health education (87.9%) dominated the home visiting activities. Health education helps to provide a safe and supportive environment and also build a strong relationship that leads to long lasting benefits to the entire family [5]. Face to face teaching in the privacy of the home is an excellent environment for imparting health information [31]. The CHNs stated that health education, tracing of defaulters, and identification of new cases are the benefits and promotion factors for conducting HVs. This implies that there are other critical aspects of HV that CHNs neglect such as prevention of home accidents and ensuring a safe home environment and care for the aged. Early detection of potential health concerns and developmental delays, prevention of child abuse, and neglect are also other benefits and promotive factors of HV. HV helps to increase parents' knowledge, parent-child interactions, and involvement [5]. The conduct of HV was not reported among all community members as some community members (22.0%) in the AD indicated their homes have never been visited. This is, however, an improvement over the rate of HVs that was reported in the Assin district in Ghana [32]. In the Assin district, about 84% of the respondents said they gained benefits from HVs [32]. In this study, respondents who were visited indicated the CHNs just inspected their weighing card while giving them no feedback. CHNs should implement various interventions to ensure that community members directly benefit from health interventions that are implemented during HVs to reduce the consequences that are usually associated with poor access to healthcare services especially in poor rural communities such as the AD.

## 5. Conclusion

The activities carried out in the homes were mainly centred on health education, contact tracing, and vaccination. Health workers faced many challenges such as geographical inaccessibility, financial constraints, and insufficient equipment and medications to treat minor ailments. If HV is carried out properly and as often as expected, one would expect the absence of home accidents, child abuse, among others in the homes, and a reduction in hospital admissions.

The need for strengthening HV as a tool for improving household health and addressing home-based management of minor ailment in the district cannot be over emphasized. It is important to forge better intersectoral collaboration at the district level. The District Assembly could assist the District Health Management Team with transport to support HVs. In addition, community-based health workers such as community health volunteers, traditional birth attendants, and community clinic attendants should also be trained to identify and address health problems in the homes to complement that which is already conducted by healthcare professionals.

## Figures and Tables

**Table 1:** Demographic characteristics of household members.

Characteristic	Frequency (*N* = 375)	Percentage (%)

Mean age (SD)	41.24 (±16.88)	

Age group in years		
<20	11	2.9
20–29	94	25.1
30–39	99	26.5
40–49	69	18.5
50–59	45	12.0
60 and above	56	15.0

Sex		
Male	93	24.9
Female	281	75.1

Religion		
Christianity	363	97.1
Islam	11	2.9

Occupation		
Business	20	6
Civil servant	26	7
Farmer	134	38
Menial jobs	32	9
Trader	86	24
Unemployed	59	17

Marital status		
Single	73	19.5
Married	262	69.9
Divorced	9	2.4
Widowed	31	8.3

Educational level		
Uneducated	46	12.3
Primary	49	13.1
JHS	176	47.2
SHS	77	20.6
Tertiary	25	6.7

**Table 2:** Practice of home visits in AD (household members).

Characteristic	Frequency	Percentage

Visit by a health worker (CHN)		
Yes	274	73.3
No	100	26.7

Last time visited by a health worker		
This week	19	7.6
Last week	40	16.1
Last month	131	52.6
Cannot remember	59	23.7

Number of times visited by a health worker(s) within the past three months		
Once	123	48.2
Twice	80	31.4
Three times	31	12.2
Had no visit	21	8.2

Time visited during the day		
9am to 2pm	255	93.4
3pm to 6pm	18	6.6

**Table 3:** Demographic characteristics of CHN.

Characteristics	Frequency (*N* = 11)	Percentage (%)

Mean age (SD)	30.44 (±4.03)	

Age		
Below 30 years	4	44.4
Above 30 years	5	55.5

Sex		
Male	1	9.1
Female	10	90.9

Religion		
Christianity	9	81.8
Islam	2	18.2

Marital status		
Single	4	36.4
Married	7	63.6

**Table 4:** Summary of CHNs home visit practice in AD.

Characteristics	Frequency	Percentage (%)
Organized home visit in the catchment area		
Yes	11	100

The ability to visit all homes in the catchment area		
Yes	9	81.8
No	2	18.2

Reason for not being able to visit all homes		
Hard to reach areas	1	50.0
Inadequate equipment	1	50.0

Type of home visiting carried out		
Only routine	3	27.3
Both (routine and special)	8	72.7

Frequency of home visits per week		
Once	1	9.1
Twice	3	27.3
Three times	5	45.5
More than three times	2	18.2

The number of homes visited in a week		
1–5	2	18.2
6–10	7	63.6
11–15	2	18.2

Times at which home visits are conducted		
Morning	10	90.9
Evening	1	9.1

Week of last home visit conducted		
This week	10	90.9
Last week	1	9.1

Health education conducted at home visit		
Yes	10	90.9
No	1	9.1

Management of minor ailment during home visit		
Yes	6	54.6
No	5	45.5

Vaccination/contact tracing during home visits		
Yes	7	63.6
No	4	36.4

## Data Availability

The data used to support the findings of this study are included within the article.

## Abbreviations

AD: Adaklu district
CHNs: Community health nurses
CWC: Child welfare clinic
CHPS: Community health planning and services
HV: Home visit
PHC: Primary healthcare
PHN: Public health nurse.
